# Association of Serum Estradiol and Testosterone With Hip Fractures in Elderly Men

**DOI:** 10.7759/cureus.101709

**Published:** 2026-01-16

**Authors:** Chiranth Kantharaj, Shishir Murugharaj

**Affiliations:** 1 Orthopedics, Kempegowda Institute of Medical Sciences, Bangalore, IND; 2 Orthopedics, Subbaiah Institute of Medical Sciences, Shimoga, IND

**Keywords:** bone health, elderly men, estradiol, hip fracture, testosterone

## Abstract

Introduction: Hip fractures in the elderly are a major public health concern due to high morbidity, mortality, and long-term disability. While men have a lower incidence of hip fractures than women, the risk rises sharply with age, and outcomes in men are often worse. Sex steroids (especially estrogen and testosterone) play a crucial role in bone metabolism, but their contribution to osteoporosis and fracture risk in elderly men remains under-investigated, particularly in diverse populations. This prospective case-control study aimed to determine whether serum estradiol and testosterone levels differ significantly between elderly South Indian men with osteoporotic hip fractures and age-matched healthy controls.

Methods: A prospective case-control study was conducted on men over 55 years of age at a South Indian tertiary hospital (March-May 2022). Fifty men with osteoporotic hip fractures (cases) and 50 age-matched healthy male controls were recruited. Clinical data (age, body mass index, smoking status) were collected, and serum total estradiol and testosterone levels were measured using enzyme-linked fluorescent immunoassay. Overt androgen deficiency was defined as total testosterone <2 ng/mL, and possible deficiency as 2-4 ng/mL. Group comparisons were performed using independent t-tests or Mann-Whitney tests for continuous variables and Fisher’s exact test for categorical variables, with significance set at p<0.05.

Results: The majority of hip fracture cases were in the 65-75 years age group (25, 50%). Cases had a high prevalence of low sex hormones: 22 (44%) had overt androgen deficiency and 26 (52%) had possible androgen deficiency, compared to only two (4%) of controls with testosterone <2 ng/mL. Mean serum testosterone in cases was significantly lower than in controls (2.40±1.12 vs. 4.22±1.62 ng/mL, p<0.001), and mean estradiol was also lower in cases (32.07±10.08 vs 41.88±17.99 pg/mL, p=0.008). Low estradiol levels were more frequent in cases: only 21 (42%) of cases had high-normal estradiol (≥34.3 pg/mL) vs. 30 (60%) of controls. There were no significant differences in body mass index or smoking status between cases and controls (p>0.05).

Conclusion: Elderly men with hip fractures had significantly lower serum testosterone and estradiol levels than age-matched controls, demonstrating an association between reduced sex hormone levels and hip fracture status. These findings support a possible role of sex hormones in male skeletal health, but causal relationships and therapeutic implications cannot be inferred from this study and require confirmation in larger prospective investigations.

## Introduction

Hip fractures represent a significant global public health concern among the elderly due to their high incidence and associated morbidity and mortality [1]. The consequences of hip fracture extend beyond the immediate injury, often leading to prolonged functional impairment, reduced mobility, and diminished quality of life [2]. This issue is compounded by the globally aging population, which is expected to substantially increase fracture prevalence and related healthcare costs [3].

While women historically exhibit a higher lifetime risk of hip fracture (approximately 18% vs 6% in men), the incidence in both sexes escalates significantly with advancing age [4]. Hip fractures in men are associated with a substantial reduction in survival, with a notable proportion of excess deaths occurring within the first six months post-fracture [5]. Mortality risk is particularly high in men over 80 years of age, underscoring the need for effective prevention and management strategies [6]. Notably, outcomes after osteoporotic hip fractures tend to be worse for men and for non-Caucasian populations compared with Caucasian women [7]. This demographic disparity in outcomes necessitates a deeper investigation into the underlying biological and environmental factors contributing to skeletal fragility in elderly men [8].

Although osteoporosis has traditionally been considered a “women’s disease” due to its higher prevalence in postmenopausal women, the absolute number of men affected by osteoporosis and fractures is substantial [9]. However, the recognition and management of osteoporosis in men have historically lagged, leading to underestimation of its impact on male health and an urgent need for more targeted research [10]. A comprehensive understanding of the interplay between sex hormones and bone health in elderly men is crucial for developing effective diagnostic and therapeutic interventions [11].

Men share some common mechanisms of age-related bone loss with women, such as accelerated bone turnover and microarchitectural deterioration, but the role of sex steroids, particularly estrogen, in male skeletal health has historically been underappreciated. Age-related hormonal changes in men include a shift from osteoblastogenesis to adipogenesis in the bone marrow, which exerts a lipotoxic effect on bone matrix formation and mineralization [12]. This complex interplay of factors underscores the necessity of investigating the specific contributions of sex hormones, especially estradiol and testosterone, to bone health and fracture susceptibility in aging males [13]. Consequently, this prospective case-control study aimed to determine whether serum estradiol and testosterone levels differ significantly between elderly South Indian men with osteoporotic hip fractures and age-matched healthy controls.

## Materials and methods

Study design and participants

This prospective case-control study was conducted in the Department of Orthopedics at a tertiary care teaching hospital in South India between March and May 2022. Institutional Ethics Committee approval was obtained prior to study initiation (IEC-SUIMS/12/2021-22), and written informed consent was obtained from all participants.

The study included 50 consecutive men aged >55 years who presented with fragility hip fractures (convenience sampling), defined as fractures occurring after low-energy trauma, such as a fall from standing height or less, and confirmed radiologically (cases). To minimize selection bias, consecutive eligible patients presenting during the study period were enrolled.

An equal number of age-matched healthy male controls (n=50) were recruited from the community and orthopedic outpatient clinics. Controls had no history of low-trauma fractures and were free of known metabolic bone disease. Matching was performed approximately for age (±3 years) and geographic region.

Eligibility criteria

Exclusion criteria for both cases and controls included pathological fractures, high-energy trauma, chronic kidney disease (stage ≥3), chronic liver disease, known thyroid or parathyroid disorders, malignancy, chronic inflammatory or autoimmune disorders, prolonged immobilization, long-term corticosteroid use, prior androgen therapy, use of anti-osteoporotic medications, and acute systemic illness at the time of sampling.

Clinical and demographic assessment

All participants underwent a standardized physical examination. Demographic and clinical data were recorded using a pre-designed proforma and included age, height, weight, body mass index (BMI), and smoking status, as these variables are recognized risk factors for osteoporosis and fractures in men. Pre-injury ambulatory status, physical activity levels, vitamin D status, sex hormone-binding globulin (SHBG), and bone mineral density (BMD) measurements were not assessed due to logistical constraints and were therefore not included in the analysis.

Hormone measurements

Fasting venous blood samples were obtained from both cases and controls; however, owing to the acute presentation of hip fracture cases, blood collection times could not be standardized. Serum total estradiol and testosterone levels were measured using an enzyme-linked fluorescent immunoassay (ELFA) technique. Total estradiol was measured with the VIDAS Estradiol II assay (bioMérieux), which has a measuring range of 9-3000 pg/mL and an inter-assay coefficient of variation of approximately 4%. Total testosterone was measured with the VIDAS Testosterone II assay (bioMérieux), with a measuring range of 0.05-13.50 ng/mL and an inter-assay coefficient of variation of approximately 4%. All assays were performed in the same laboratory using standardized protocols to minimize analytical variability.

According to Endocrine Society guidelines (USA and Australia), a total testosterone level <2 ng/mL is considered indicative of overt androgen deficiency, while levels in the range of 2-4 ng/mL suggest possible androgen deficiency. There are currently no established cutoff levels below which estradiol is considered deficient in men; thus, estradiol levels were analyzed as a continuous variable and also categorized into tertiles for exploratory analysis.

Statistical analysis

All data were assessed for normality prior to analysis. Continuous variables are presented as mean ± standard deviation, and categorical variables as counts and percentages. Group comparisons between cases and controls were performed using the independent Student’s t-test for approximately normally distributed continuous data and the Mann-Whitney U test for non-parametric data. Categorical variables were compared using Fisher’s exact test, and odds ratios (ORs) with 95% confidence intervals were calculated to assess the strength of association between risk factors (such as low hormone levels or smoking) and hip fracture status. A two-tailed p-value <0.05 was considered statistically significant. Data analysis was conducted using IBM SPSS Statistics for Windows, version 23.0 (IBM Corp., Armonk, NY, USA).

## Results

Out of the 50 elderly men with hip fractures (cases), the majority, 25 (50%), were in the 66-75 year age group, reflecting the older age distribution of fracture patients (Table 1). The age distribution of the control group was similar, as they were age-matched, with a similarly high proportion in the seventh decade of life.

**Table 1:** Age distribution among cases and controls

Age (years)	Cases	Controls
55-65	12 (24%)	11 (22%)
66-75	25 (50%)	21 (42%)
76-85	9 (18%)	10 (20%)
85-95	4 (8%)	8 (16%)
Total	50 (100%)	50 (100%)

A striking finding was the high prevalence of low sex hormone levels among the hip fracture cases, as compared to controls. In the case group, 22 out of 50 men (44%) exhibited overt androgen deficiency (total testosterone <2 ng/mL) and an additional 26 (52%) had testosterone levels in the 2-4 ng/mL range (possible deficiency). By contrast, the vast majority of controls had testosterone levels above 4 ng/mL; only 2 controls (4%) fell below 2 ng/mL (Table 2).

**Table 2:** Serum testosterone level categories among cases and controls

	Testosterone groups (range of testosterone levels)		
Serum testosterone levels	Low (<2 ng/mL)	Middle (2-4 ng/mL)	High (>4 ng/mL)
Cases (50)	22 (44%)	26 (52%)	2 (4%)
Controls (50)	2 (4%)	23 (46%)	25 (50%)

Similarly, serum estradiol levels tended to be lower in cases. When categorized into tertiles, only 4 cases (8%) had estradiol levels in the low range (<18.2 pg/mL), and 21 (42%) had high-range estradiol levels (≥34.3 pg/mL), whereas 30 controls (60%) had high estradiol levels, and very few controls had low estradiol levels (Table 3). These distributions suggest that men with hip fractures were more likely to have deficiencies in both testosterone and estradiol compared with their healthy counterparts.

**Table 3:** Serum estradiol level categories among cases and controls

	Estradiol groups (range of estradiol levels)		
Serum estradiol levels	Low (2-18.1 pg/mL)	Middle (18.2-34.2pg/mL)	High (≥34.3 pg/mL)
Cases (50)	4 (8%)	25 (50%)	21 (42%)
Controls (50)	2 (4%)	18 (36%)	30 (60%)

Direct comparison of mean hormone concentrations confirmed these differences. The mean total testosterone level in cases was 2.40±1.12 ng/mL, significantly lower than 4.22±1.62 ng/mL in controls (p<0.001). The chi-square test was used to compare cases and controls, and a p-value <0.05 was considered statistically significant (Table 4). Likewise, the mean estradiol level in cases was 32.07±10.08 pg/mL versus 41.88±17.99 pg/mL in controls, representing a statistically significant difference (p=0.008). The Mann-Whitney U test was used to compare cases and controls, with a p-value <0.05 considered statistically significant (Table 4). These data indicate that men who sustained hip fractures had substantially lower circulating testosterone and estradiol levels on average compared with age-matched men without fractures.

**Table 4:** Serum testosterone, estradiol, and BMI among study groups

Parameter	Group	Mean	Standard deviation	P-value	95% CI
BMI (kg/m²)	Cases	24.43	3.69	0.508	23.38-25.48
	Controls	24.93	3.80		23.85-26.01
Serum testosterone	Cases	2.40	1.12	<0.001	2.08-2.72
	Controls	4.22	1.62		3.76-4.68
Serum estradiol	Cases	32.07	10.08	0.008	29.20-34.93
	Controls	41.88	17.99		36.77-46.99

Apart from differences in sex hormone levels, there were no significant differences in BMI or smoking status between the case and control groups. The mean BMI of fracture cases was 24.43±3.69, which was comparable to 24.93±3.80 in controls (Table 4), with p=0.508 indicating no statistical significance.

Similarly, the distribution of smokers versus non-smokers did not differ appreciably: 12 (24%) of cases were current smokers compared with 17 (34%) of controls, and this difference was not statistically significant (chi-square p=0.271) (Table 5). These results suggest that, in this study population, conventional risk factors such as BMI and smoking were not strongly associated with hip fracture risk when comparing cases with controls.

**Table 5:** Smoking status among cases and controls

	Non-smoker	Smoker	Total	P-value
Cases	38 (76%)	12 (24%)	50	0.271
Controls	33 (66%)	17 (34%)	50	
Total	71	29	100	

## Discussion

The finding that the majority of hip fracture cases were observed in men aged 66-75 years aligns with epidemiological data suggesting an increasing incidence of osteoporotic fractures with advancing age, particularly in older male populations [14]. This age-related vulnerability to fractures is compounded by physiological changes in bone quality and architecture that occur with senescence, increasing susceptibility to low-trauma fractures [15,16]. This demographic trend underscores the importance of targeted screening and intervention strategies for older men to mitigate fracture risk [17,18]. Furthermore, the concentration of fracture events within a relatively narrow age bracket (late 60s to mid-70s) may indicate an inflection point at which age-related bone density decline and hormonal changes collectively heighten fracture susceptibility, necessitating early identification of at-risk individuals [14].

The significant prevalence of overt and possible androgen deficiency among the hip fracture cases (44% and 52% of cases, respectively) strongly indicates an association between diminished serum testosterone levels and increased susceptibility to hip fractures in elderly men. This finding is consistent with existing literature implicating male hypogonadism in reduced BMD and higher fracture risk [19,20]. Conversely, the observed association between low serum estradiol levels and hip fracture, despite reaching statistical significance (p=0.008), introduces a nuanced perspective. Males produce a significant portion of estradiol via aromatization of testosterone; therefore, low estradiol levels in men often reflect low testosterone levels. This interdependence suggests a complex interplay between these hormones in maintaining skeletal integrity [21]. Both low testosterone and low estradiol may synergistically contribute to compromised bone strength, and disentangling their individual effects is challenging due to their biochemical linkage.

Thus, the present findings underscore the need for focused, hypothesis-driven studies to better understand the role of sex steroids in male skeletal health rather than broad exploratory investigations. It is particularly important to understand how shifts in the testosterone-to-estradiol ratio influence bone remodeling pathways and fracture susceptibility. Further research is needed to elucidate the precise mechanisms through which testosterone and estradiol, independently and in combination, affect bone health in aging men, beyond their established roles in maintaining BMD [22]. Future studies should also focus on defining optimal threshold levels of serum testosterone and estradiol that are protective against fractures in elderly men. Investigating genetic polymorphisms that affect steroid hormone synthesis, metabolism, and receptor sensitivity could shed light on individual susceptibility to osteoporosis [23]. Moreover, longitudinal studies tracking changes in hormone levels and bone density over time are essential to establish a causal relationship and refine predictive models for hip fracture risk in this demographic. In addition, investigations into the role of SHBG are warranted, since variations in SHBG levels can alter the bioavailability of testosterone and estradiol, thereby potentially impacting their effects on the skeleton.

The implications of this study are confined to enhancing understanding of the hormonal milieu in elderly men with hip fractures rather than supporting therapeutic recommendations. Although existing evidence demonstrates that estradiol contributes to male skeletal maintenance and that even low circulating levels can influence bone turnover and density [24-26], the present observational findings cannot be used to infer benefit from androgen- or estrogen-modulating therapies. Any consideration of hormonal interventions for fracture prevention in men would require confirmation through adequately powered prospective and interventional studies.

Limitations

The single-center design and modest sample size limit generalizability and causal inference, and hormone levels were assessed at a single, non-standardized time point. Additionally, unmeasured factors such as BMD, vitamin D status, SHBG levels, and pre-injury functional status may have contributed to residual confounding.

## Conclusions

In summary, this study shows that elderly South Indian men with hip fractures have significantly lower serum testosterone and estradiol levels compared with age-matched controls, indicating an association between reduced sex hormone levels and hip fracture status. These findings suggest a potential role for testosterone and estrogen in male skeletal health; however, given the observational design, they should be interpreted cautiously and not as evidence of causality or therapeutic benefit. Further well-designed prospective studies are needed to clarify the underlying mechanisms and to determine the clinical relevance of hormonal assessment in fracture risk evaluation among elderly men.
